# Metacognitive beliefs as psychological predictors of social functioning: An investigation with young people at risk of psychosis

**DOI:** 10.1016/j.psychres.2017.09.037

**Published:** 2018-04

**Authors:** Measha Bright, Sophie Parker, Paul French, David Fowler, Andrew Gumley, Anthony P. Morrison, Max Birchwood, Peter B. Jones, Suzanne L.K. Stewart, Adrian Wells

**Affiliations:** a School of Health Sciences, Division of Psychology & Mental Health, The University of Manchester, Manchester, United Kingdom; b Greater Manchester Mental Health NHS Foundation Trust, Manchester, United Kingdom; c School of Psychology, University of Sussex, Brighton, United Kingdom; d Sussex Partnership NHS Foundation Trust, Hove, United Kingdom; e University of Glasgow, United Kingdom; f Warwick Medical School, University of Warwick, United Kingdom; g Department of Psychiatry, University of Cambridge, United Kingdom; h Department of Psychology, University of Chester, United Kingdom

**Keywords:** ARMS, At Risk Mental State, Metacognition, Structured activity, Cognitive, ARMS, Schemas, Positive symptoms, Social anxiety, Depression

## Abstract

Poor social functioning has been found to be present in those at risk for psychosis. This study aimed to examine metacognitive beliefs as potential predictors of structured activity (measure of social functioning) in those with an At Risk Mental State (ARMS). Regression and correlation analyses were conducted. The sample included 109 young people. Age was found to be positively correlated to structured activity. Metacognitive beliefs concerning uncontrollability and danger of worry were found to negatively predict structured activity. This was after controlling for age, gender, treatment allocation, cognitive schemas, positive symptom severity, social anxiety, and depression. Metacognitive danger items were most important. Age was the only control variable found to be an independent predictor of structured activity in the regression model, despite negative bi-variate relationships with structured activity found across three cognitive schema subscales and social anxiety. This is the first study to find that higher negative metacognitive beliefs about uncontrollability and danger predict lower social functioning in an ARMS sample, and that the perception of thoughts being dangerous was of particular importance. Psychological interventions should consider targeting this metacognitive dimension to increase social functioning. Future longitudinal research is required to strengthen findings in this area.

## Highlights

• Age is a positive predictor of structured activity.
• A single negative metacognitive belief negatively predicted structured activity.
• Dangerousness of thoughts was an important predictor of structured activity.
• More research is needed in this area and of a longitudinal nature.

## 1 Introduction

Poor functioning has been found to be present prior to the onset of psychosis and as such, is included in the criteria for identifying those with an ‘At Risk Mental State’ (ARMS) (Cannon et al., 2008, Yung et al., 2004, Yung et al., 2005). Social functioning specifically has received increased attention in at risk for psychosis research. The definition of social functioning varies across research in this area. Based on the measures used to assess social functioning in past research with young people with an ARMS, this construct tends to relate to occupational and educational performance, relationships with peers and family members, engagement in leisure and sports activities, level of independence and interpersonal and communication abilities (Addington et al., 2008, Addington et al., 2013, Ballon et al., 2007, Cornblatt et al., 2007, Hodgekins et al., 2015, O'Brien et al., 2006, Palmier-Claus et al., 2016, Rapado-Castro et al., 2015).

Social functioning (measured with the Time Use Survey) has been found to be significantly lower in those with an ARMS and those experiencing psychosis than in the non-clinical population (Hodgekins et al., 2015). The Time Use Survey measures structured activity (i.e. education, employment, leisure and sports activity, childcare and housework and chores). Hodgekins et al. (2015) identified that 50% of those with an ARMS engaged in 30 h or less of structured activity per week. Participants scoring 45 h or less per week on this measure were considered to be ‘socially disabled’ (significantly lower social functioning scores than the non-clinical population) and scoring within clinical parameters.

Past research has identified social functioning to be both a ‘trait’ and ‘state’ factor in those at risk of schizophrenia (Shim et al., 2008). However, Shim et al. (2008) found that positive and negative symptoms did not have significant relationships with social functioning. However, significant relationships between social functioning and depressive, negative and disorganised symptoms, but not positive symptoms in young people at risk for psychosis have been reported (Corcoran et al., 2011). Chudleigh et al. (2011) in a study of the early stages of psychosis found significant relationships between positive symptoms and qualitative (but not quantitative) measures of social functioning in those at risk for and experiencing a first episode psychosis. No significant relationships were found between social functioning and negative symptoms. They also reported large significant associations between depression and quantitative and qualitative measures of social functioning in those at risk for psychosis. Social anxiety did not have any relation to social functioning in those at risk for psychosis, but large correlations were found between these variables in the first episode psychosis group (Chudleigh et al., 2011). It appears that the relationship between social functioning and symptomatology is a complex one in those experiencing early psychosis. Social functioning difficulties are known to be a source of distress for young people experiencing them, above and beyond psychotic and depressive symptoms (Rapado-Castro et al., 2015). More work needs to be done to establish the factors related to social functioning in those experiencing psychosis.

From the perspective of psychological research and intervention, cognitive therapy approaches have focused on negative beliefs (cognitive schemas) as a key area of investigation and are an integral element in some cognitive models of psychosis (Garety et al., 2007). Negative beliefs about the self and others were found to be significantly higher in clinical groups (ARMS, first episode psychosis and a help-seeking psychosis group) than in non-clinical controls (Taylor et al., 2014). Another study found high ratings of the same negative schemas to be significantly related to lack of trust and social isolation, whilst positive beliefs about the self and others were significantly linked to reduced levels of social isolation (Addington and Tran, 2009). This indicates a potential relationship between negative schemas and social functioning.

However, recent work has begun to question the primacy of cognitive schemas in psychopathology, and metacognition (broadly defined as thinking about thinking) has become a focus of investigation (Wells, 2009). In the Self-Regulatory Executive Function (S-REF) model, (Wells and Matthews, 1994, Wells and Matthews, 1996), a metacognitive model of psychological disorders, dysfunction is thought to be caused by repetitive negative thinking that is difficult to bring under control as well as increased self-focussed attention. This Cognitive Attentional Syndrome (CAS) consists of rumination, worry, threat monitoring and engagement in unhelpful coping strategies (e.g. avoidance of others, thought suppression, substance misuse). The CAS is hypothesised to be linked to underlying knowledge about cognition (i.e. metacognitive beliefs) and therefore metacognitive beliefs rather than cognitive schemas are considered to be predominant contributors to the development and maintenance of psychological disorder. According to the model there are two main types of metacognitive beliefs, positive beliefs and negative beliefs. Measures have been developed to assess such metacognitive beliefs, the primary one being the metacognitions questionnaire-30 (MCQ-30) (Wells and Cartwright-Hatton, 2004). This measure assesses five dimensions of metacognitive beliefs. Positive beliefs about worry which concerns the benefits to engaging in worry (e.g. *‘Worrying helps me cope.’);* negative beliefs about uncontrollability of thoughts and danger which relate to the perceived dangerousness of thoughts (e.g. *‘I could make myself sick with worrying.’)*; cognitive confidence (e.g. *‘I have a poor memory.’*); negative beliefs about the need to control thoughts (e.g. *‘If I did not control a worrying thought, and then it happened, it would be my fault.’)*; and cognitive self-consciousness (e.g. *‘I monitor my thoughts.’*).

Consistent with this theory, past research has identified unhelpful metacognitions to be present in those experiencing depression (Papageorgiou and Wells, 2001, Wells et al., 2009), anxiety (Wells and King, 2006), and psychosis (Austin et al., 2014, Morrison et al., 2007, Morrison and Wells, 2007, Sellers et al., 2016). A related concept of meta-worry (Wells and Matthews, 1994), which consists of worry about worry has also been found to be positively associated with delusional distress (Freeman and Garety, 1999). Also change in meta-worry appears to correlate with symptom change in people undergoing cognitive therapy for psychosis (Parker et al., 2014). Unhelpful metacognitions have also been identified as being present in those at risk for psychosis (Barbato et al., 2014, Cotter et al., 2017, Morrison et al., 2007, Welsh et al., 2014). Cotter et al. (2017) conducted a systematic review and meta-analysis of metacognitive beliefs in those at risk for psychosis and found that those with an ARMS had significantly elevated scores (p < 0.001) on measures of metacognitive beliefs compared to healthy controls. This was true for all metacognitive subscales except positive beliefs about worry (p = 0.053). No significant differences were found between those with an ARMS and those experiencing psychosis. This past research provides evidence of the presence of unhelpful metacognitive beliefs in those at risk for psychosis. However, no research to date has explored how these metacognitions, as described by the Wells and Matthews model, affect social functioning in young people with ARMS. This is an important area because metacognitions, especially those related to uncontrollability or danger of thinking, might impact on activity levels and represent a common factor contributing to both risk and reduced activity.

This study aims to explore the role of metacognitive beliefs in predicting social functioning in those at risk for psychosis. Although there is no past research on the effects of metacognitive beliefs on social functioning specifically, there is an increasing amount of research identifying the presence of maladaptive metacognitions in a range of psychological disorders including ARMS. Further, the S-REF model suggests that metacognitive beliefs are linked to unhelpful coping strategies as typified by the CAS, such as increased worry and avoidance. Coping in this way is likely to lead to reduced social contact, and if persistent over time social isolation. It was predicted, therefore, that metacognitive beliefs will be negatively related to social functioning. However, the paucity of research in this specific area means that it is difficult to make specific predictions about which metacognitions might be involved. Due to this, we kept our hypothesis broad and investigated all of the metacognitive beliefs as measured by the MCQ-30. This study controlled for age, gender, cognitive schemas and symptoms to assess the contribution of metacognitive beliefs in predicting structured activity.

## 2 Methods

### 2.1 Participants

Data used for this study were drawn from measures administered with participants identified as being at risk for psychosis in the Early Detection and Intervention Evaluation 2 (EDIE-2) trial (Morrison et al., 2012). EDIE-2 was a multi-site randomised controlled trial with young people at risk for psychosis investigating the efficacy of Cognitive Therapy (CT) on reducing transition to psychosis. Participants were recruited from 5 UK sites: Manchester, Birmingham/Worcestershire, Glasgow, Cambridgeshire and Norfolk. The study recruited 288 participants (144 in each arm) aged 14–35 years. Participants were allocated to receive CT plus monitoring or monitoring alone. Monitoring involved signposting where symptoms worsened, providing helpline telephone numbers and checking participants were registered with their GP. Monitoring in both arms was conducted on a monthly basis for the first 6 months, and then once every 3 months thereafter. More detailed follow-ups were conducted at 6, 12, 18 and 24 months. CT was found not to significantly reduce transition to psychosis, but did reduce the severity of symptoms in those at risk for psychosis.

**Fig. 1:**
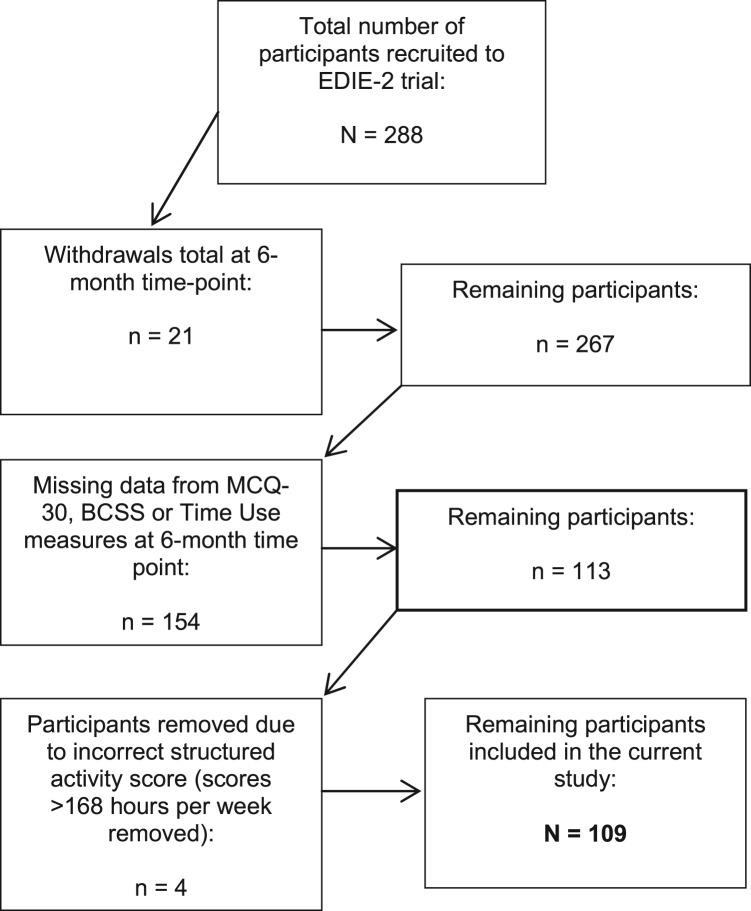
Illustration of how the participant number (N = 109) is arrived at in this study.

Data for this study were drawn from measures administered at the 6 month time-point because this was the only time-point both of the key measures required for analysis (i.e. metacognitive beliefs and social functioning measures) were administered. The total sample size for the primary analysis in this study was 109 participants rather than all 288 recruited into EDIE-2. Fig. 1 shows how the number of participants included in this study was arrived at. Participants allocated to the treatment arm of the EDIE-2 trial would have received therapy prior to completing these measures as CT was provided over the first 6 months. There was a relatively even split for those who received CT (n = 56) and those allocated to treatment as usual (n = 53). The male to female ratio was 63:46. Sixty seven per cent of those recruited into the EDIE-2 study were found to meet criteria for at least one other psychological disorder, as defined by the Structured Clinical Interview of DSM-IV Axis I Disorders (SCID). Depression (31.5%) was the most common co-morbid disorder followed by panic disorder without agoraphobia (12.5%), and then social phobia (10.42%).

### 2.2 Design and analyses

This study is cross-sectional in nature using data collected at the 6-month time-point.

Bi-variate (Pearson) correlation analyses were conducted to examine relationships between cognitive and metacognitive subscales, symptoms and structured activity. Hierarchical multiple linear regression analyses were performed testing metacognitive beliefs as predictors of structured activity, whilst controlling for age, gender, treatment allocation, positive symptoms, social anxiety, depression and cognitive schemas. All analyses were performed using IBM SPSS Statistics Version 22 (SPSS).

### 2.3 Measures and procedures

The Comprehensive Assessment of At Risk Mental States (CAARMS) is a semi-structured interview, developed by Yung et al. (2005), that assesses for psychotic symptoms and determines if individuals are at risk for psychosis. This measure can also detect if individuals meet criteria for psychosis. ARMS status is determined if participants fall into any of the following groups within the preceding 12 months of the CAARMS assessment being administered: 1) Genetic risk in a first degree relative; 2) Attenuated psychotic symptoms; or 3) Brief Limited Intermittent Psychotic Symptoms (BLIPS) that resolve within a week without antipsychotic medication. Individuals will also have to score 50 or less (1 = very poor general functioning (e.g. severe attempts on ending own life) and 100 = excellent general functioning (e.g. involved in a variety of activities)) on the Global Assessment of Functioning scale (GAF) in the past month or have a drop in functioning by 30% or more in the last 12 months. It is worth noting that the version of the CAARMS in the EDIE-2 trial used the GAF to measure general functioning, rather than the updated 2014 version that uses the Social and Occupational Functioning Assessment Scale (SOFAS) which is a purer measure of social functioning as it does not include symptomology (e.g. anxiety or mood) in the scoring. The CAARMS used in the current study has been found to have ‘good to excellent reliability’ (Yung et al., 2005, p.964).

The Time Use Survey (created by the Office for National Statistics for a study exploring the Time Use of the general population (Lader et al., 2005)) was used to measure social functioning in the form of structured activity in the EDIE-2 trial. Structured activity in this measure is defined as time spent in paid employment, education, voluntary work, leisure and sports activities, child care, and housework and chores. The Time Use Survey covers several of the areas earlier identified as being measures of social functioning in past research. Weekly hourly scores were calculated for each participant in the EDIE-2 trial by asking about structured activity in the last 3 months. This quantitative measurement of structured activity allows social functioning to be measured across participants. Structured activity at the 6 month time point will be the focus of this investigation.

The Meta-Cognitions Questionnaire-30 (MCQ-30) (Wells and Cartwright-Hatton, 2004) is a 30-item self-report questionnaire. It measures five dimensions of metacognitive beliefs about worry and thoughts as well as judgements about thinking and it has been found to have good reliability and validity (Wells and Cartwright-Hatton, 2004). Cronbach alphas were calculated for the MCQ-30 in the current study and all sub-scales had high reliability with Cronbach alphas in excess of 0.8. Positive beliefs about worry α = 0.93; negative beliefs about uncontrollability and danger α = 0.90; cognitive confidence α = 0.89; negative beliefs about the need to control thoughts α = 0.81; and cognitive self-confidence α = 0.88.

The Beliefs about the Self and Others (BCSS) (Fowler et al., 2006) is a 24-item self-report questionnaire that was designed to measure cognitive schemas in psychosis. Four schemas are measured: positive beliefs about the self; negative beliefs about the self; positive beliefs about others; and negative beliefs about others. Internal consistency has been found to be reliable (Fowler et al., 2006) and appropriate (Addington and Tran, 2009) in the ARMS population. Cronbach alphas for the current study were more than 0.8 illustrating high reliability. Negative beliefs about self α = 0.86; negative beliefs about others α = 0.92; positive beliefs about self α = 0.88; and positive beliefs about others α = 0.94.

The Beck Depression Inventory-7 (BDI7) is a brief 7-item self-report measure used to assess level of depression. Past research has found this measure to be highly reliable and valid (Beck et al., 1997). Reliability was also high for the data in this study with a Cronbach alpha of 0.91.

The Social Interaction Anxiety Scale (SIAS) 20-item self-report measure of social anxiety found to be reliable and valid (Mattick and Clarke, 1998). The SIAS measures worries about general social interactions, and items are linked to the DSM-III-R criteria for social phobia (Mattick and Clarke, 1998). Cronbach alpha of 0.93 showed high reliability in this study,

Research assistants who were fully trained in administering all the measures collected the data in the EDIE-2 trial. A more comprehensive description of the study procedures can be found in Morrison et al. (2012).

## 3 Results

### 3.1 Descriptive statistics

Descriptive statistics for age, structured activity, GAF, CAARMS symptom severity, SIAS, BDI, BCSS and MCQ-30 scores at 6 month time point are shown in Table 1. The number of participants included in the analyses with an ARMS was 106, as defined by the CAARMS criteria (symptoms met within 12 months). The other three participants met criteria for psychosis. Forty-five of the ARMS participants were experiencing current (within last month) ARMS symptoms at the 6 month time-point.

**Table 1:** Descriptive statistics.

Factor	N	Minimum	Maximum	Mean	Standard Deviation
Age	109	14.00	34.00	20.71	4.34
Structured activity	109	0.08	126.31	39.21	27.94
GAF	109	10	90.00	60.19	15.92
*CAARMS symptom severity:*					
Unusual thought content (UTC)	109	0	6.00	1.43	1.86
Non-bizarre ideas (NBI)	109	0	6.00	1.75	1.73
Perceptual abnormalities (PA)	109	0	5.00	1.39	1.66
Disorganised speech (DS)	109	0	4.00	1.08	1.25
BDI Total	105	0	18.00	5.36	4.68
SIAS Total	104	0	73.00	30.75	18.07
*Schemas:*					
BCSS Negative self	109	0	22.00	5.63	5.60
BCSS Negative other	109	0	24.00	7.57	6.36
BCSS Positive self	109	0	24.00	8.07	6.10
BCSS Positive other	109	0	24.00	9.44	6.39
*Metabeliefs:*					
Cognitive confidence	109	6	24.00	11.92	4.70
Positive beliefs about worry	109	6	24.00	10.40	4.67
Cognitive self-consciousness	109	6	24.00	14.98	4.90
Negative uncontrollability and danger	109	6	24.00	14.30	5.47
Beliefs about thought control	109	6	23.00	11.79	4.27
MCQ-30 Total	109	30.00	105.00	63.39	17.27
Danger Total	109	3.00	12.00	6.72	2.87
Uncontrollability Total	109	3.00	12.00	7.58	3.02

### 3.2 An examination of the relationship between metacognitive beliefs, cognitive schemas, age, gender, symptoms and structured activity

Pearson correlations were conducted to examine the inter-relationship between measures. The coefficients are presented in Table 2. There was a moderate positive relationship between age and structured activity. Small negative relationships existed between both negative cognitive schema subscales and structured activity. A small positive correlation was present between the positive beliefs about self cognitive schema subscale and structured activity. No significant relationship was found between positive beliefs about others and structured activity. No significant relationships existed between any of the CAARMS symptom severity subscales or the BDI-7 and structured activity. A small negative relationship was found between the SIAS score and structured activity. One metacognitive belief subscale, negative beliefs about uncontrollability and danger, had a small negative relationship to structured activity. This subscale was broken down into its two parts (danger and uncontrollability). Danger and uncontrollability each had small negative relationships with structured activity.

**Table 2:** Correlation matrix for structured activity at 6 months, cognitive and metacognitive beliefs, age and gender (N = 100).

	2	3	4	5	6	7	8	9	10	11	12	13	14	15	16	17	18	19	20	21
1. Structured activity 6 months	0.34^**^	− 0.02	0.11	− 0.06	− 0.19	− 0.08	− 0.06	− 0.11	− 0.21*	− 0.22*	− 0.24*	0.21*	0.11	− 0.11	− 0.08	− 0.10	− 0.22*	− 0.13	− 0.20*	− 0.21*
2. Age	–	− 0.03	0.15	− 0.06	0.08	0.08	− 0.12	0.23*	− 0.12	0.12	− 0.11	0.11	0.06	− 0.13	− 0.13	0.09	0.11	0.02	0.13	0.08
3. Gender		–	− 0.08	− 0.07	0.05	0.12	− 0.17	− 0.09	− 0.02	0.11	− 0.03	0.08	0.11	0.08	− 0.03	− 0.03	0.25*	− 0.01	0.19	0.27**
4. Treatment allocation			–	− 0.07	0.01	− 0.12	0.02	0.05	0.00	0.04	0.05	− 0.11	− 0.05	− 0.01	0.21*	0.10	0.02	0.05	− 0.04	0.08
5. Unusual thought content severity				–	0.51**	0.47**	0.34**	0.30**	0.18	0.27**	0.16	0.04	0.02	0.18	0.07	0.15	0.26*	0.30**	0.28**	0.20*
6. Non-bizarre ideas severity					–	0.32**	0.20*	0.43**	0.33**	0.41**	0.22*	− 0.08	0.02	0.24*	0.19	0.33**	0.36**	0.43**	0.33**	0.34**
7. Perceptual abnormalities severity						–	0.08	0.17	0.12	0.22*	0.00	0.05	− 0.07	0.07	− 0.10	0.06	0.05	0.26**	0.00	0.10
8. Disorganised speech severity							–	0.05	0.20*	0.13	0.18	0.06	0.00	0.12	0.03	0.00	0.01	0.08	0.06	− 0.04
9. BDI-7 Total								–	0.33**	0.63**	0.38**	− 0.23*	− 0.24*	0.27**	0.19	0.44**	0.48**	0.43**	0.43**	0.48**
10. SIAS Total									–	0.52**	0.35**	− 0.36**	− 0.33**	0.46**	0.36**	0.30**	0.38**	0.40**	0.35**	0.35**
11. BCSS Negative self										–	0.43**	− 0.37**	− 0.24*	0.36**	0.23*	0.36**	0.45**	0.41**	0.43**	0.41**
12. BCSS Negative other											–	− 0.15	− 0.24*	0.33**	0.30**	0.25*	0.35**	0.46**	0.31**	0.34**
13. BCSS Positive self												–	0.65**	− 0.28**	− 0.08	− 0.07	− 0.12	− 0.23*	− 0.05	− 0.16
14. BCSS Positive other													–	− 0.18	− 0.21*	− 0.18	− 0.19	− 0.24*	− 0.15	− 0.21*
15. Cognitive confidence														–	0.31**	0.33**	0.19	0.40**	0.16	0.18
16. Positive beliefs about worry															–	0.52**	0.32**	0.54**	0.25*	0.34**
17. Cognitive self-consciousness																–	0.55**	0.53**	0.54**	0.49**
18. Negative beliefs uncontrollability and danger																	–	0.46**	0.93**	0.93**
19. Beliefs about thought control																		–	0.04**	0.46**
20. Danger total																			–	0.73**
21. Uncontrollability total																				–

Correlation significance levels: *0.05 level, **0.01 level.

### 3.3 Do metacognitive beliefs predict structured activity after controlling for age, gender, treatment allocation and cognitive schemas?

A mixed hierarchical multiple regression was run to establish whether metacognitive beliefs predicted social functioning outcome in those at risk for psychosis, controlling for the following variables: age, gender, treatment allocation and cognitive schemas (N = 109). Age and gender were entered at step 1 of the model using forced entry. Age but not gender significantly predicted structured activity, multiple *R* was 0.09 F(2,106) = 5.28, p < 0.01. The adjusted R^2^ was 0.07 indicating a small amount of variance could be explained by these predictor variables. Treatment factors were entered at step 2 and cognitive schemas at step 3 using forced entry. No significant relationships to structured activity were found for any of these variables with only age remaining significant at each step. Metacognitive beliefs were specified at step 4 and as there is a lack of past research in the area of metacognition and social functioning, the forward selection option was chosen to determine the strongest individual predictors. Negative beliefs about uncontrollability and danger was found to be a negative predictor of structured activity, R square change = 0.04, F change = 4.51, p = 0.04.

As negative beliefs about uncontrollability and danger of thoughts entered the model, uncontrollability and danger items that constitute this factor were examined separately to extract more data on specific predictors of structured activity. The regression was run again exactly as described above, but instead of including all metacognitive belief subscales at step 4, the danger and uncontrollability sub-sets of items were entered instead. Danger, but not uncontrollability was found to be a negative predictor of structured activity, R square change = 0.03, F change = 4.11, p = 0.045. The summary statistics for each step in the equation of this model are displayed in Table 3.

**Table 3:** Output for all steps of the regression model predicting structured activity using separate danger and uncontrollability totals in step 4 controlling for cognitive schemas, age, gender and treatment allocation (N = 114).

	∆*R*	∆*F*	*p*	β	t	*p*
*Step 1*	0.09	5.62	0.005			
Age				0.30	3.353	0.001
Gender				0.02	0.17	0.864
*Step 2*	0.00	0.05	0.821			
Age				0.30	3.30	0.001
Gender				0.02	0.18	0.861
Treatment allocation				0.02	0.23	0.821
*Step 3*	0.07	2.25	0.069			
Age				0.25	2.70	0.008
Gender				0.03	0.34	0.738
Treatment allocation				0.07	0.74	0.462
BCSS Negative beliefs about others				− 0.08	− 0.70	0.487
BCSS Positive beliefs about self				− 0.15	− 1.40	0.166
BCSS Negative beliefs about self				0.17	1.33	0.185
BCSS Positive beliefs about others				− 0.03	− 0.26	0.797
*Step 4*	0.03	4.11	0.045			
Age				0.28	2.96	0.004
Gender				0.06	0.69	0.493
Treatment allocation				0.06	0.66	0.510
BCSS Negative beliefs about others				− 0.01	− 0.06	0.956
BCSS Positive beliefs about self				− 0.12	− 1.08	0.282
BCSS Negative beliefs about self				0.19	1.54	0.127
BCSS Positive beliefs about others				− 0.06	− 0.48	0.634
MCQ-30 Danger				− 0.21	− 2.03	0.045

### 3.4 Do metacognitive beliefs still continue to contribute to structured activity when also controlling for CAARMS symptom severity, social anxiety and depression?

A further mixed hierarchical regression was run to test if metacognitive beliefs continued to contribute to structured activity when also controlling for symptoms (N = 100). Age and gender were entered at step1, treatment allocation at step 2, CAARMS symptom severity at step 3, SIAS and BDI-7 at step 4 and cognitive schemas at step 5 all using forced entry. Metacognitive beliefs were entered at the final step (step 6) using forward entry. Age continued to significantly predict SA, multiple *R* was 0.12 F(2,97) = 6.52, p < 0.01. The adjusted R^2^ was 0.10. Negative beliefs about uncontrollability and danger also continued to contribute to structured activity when controlling for symptoms, R square change = 0.03, F change = 4.05, p = 0.047. Danger remained a predictor when re-running this analysis as described above, but including danger and uncontrollability sub-subscales instead of metacognitive beliefs in the model at step 6 (N = 104). The R square change was 0.03, F change = 4.17, p = 0.044.

Multicollinearity was not an issue in any of the regression models. None of the inter-correlation coefficients were higher than 0.80 suggesting none of the variables in the models measured the same construct. Further, the Variance Inflation Factor (VIF) statistics for all variables were less than 10 (highest VIF out of all models = 2.56) and all Tolerance figures more than 0.20 (lowest Tolerance figure = 0.39) providing additional confirmation that multicollinearity was not present between any of the variables. The Durbin-Watson values were within acceptable parameters (ranging between 2.02 and 2.56 across all models) indicating that no autocorrelations were made to the residuals in the models. Normality of residuals was tested using a histogram and normal P-P plot. The residuals were normally distributed. Scatter plots showed the assumptions of homoscedasticity to be met.

## 4 Discussion

Examination of the psychological predictors of structured activity could provide a useful step forward in understanding variation in structured activity and provide a means of linking it meaningfully with risk. In this study we examined the role of metacognitive beliefs, whilst controlling for other factors that could also contribute to social functioning outcome.

Consistent with our predictions, metacognitive belief was found to be a negative correlate of structured activity. A single metacognitive belief subscale, negative beliefs about uncontrollability of thoughts and danger, predicted structured activity over and above control variables. The findings show that the higher the score on this subscale, the lower the social functioning. This was the case after controlling for age, gender,treatment allocation,positive symptom severity, social anxiety, depression and cognitive schemas. Due to the lack of past research exploring the effects of metacognitive beliefs on social functioning, we were unsure which subscales would be predictors in the regression model. These results give us a better idea of specifically which metacognitive beliefs may need to be targeted and examined further.

Although three of the four cognitive schemas correlated with social functioning, when added to the regression model none of the cognitive schema subscales predicted social functioning. Social anxiety was the only symptom to correlate with structured activity. None of the symptoms included in the regression model were found to predict structured activity.

These data are consistent with the idea that structured activity is a marker for maladaptive metacognitive beliefs concerning the dangerousness and (possible) uncontrollability of one's thoughts. Such metacognitions have been causally linked to the development of psychological disorder (Wells, 2009). Therefore, an important possibility is that specific metacognitions could account for both greater at risk status and reduced social activities. Why should someone with such metacognitions show reduced social functioning? It is likely that believing that one's thoughts are dangerous leads to avoidance of situations that may provoke negative thoughts in an attempt to keep oneself and other's safe. Reduced social functioning releases the individual from having to constantly monitor and control thinking to prevent threat. However, this must remain highly speculation as the design of this study does not allow for the testing of causal relationships.

Age was found to significantly predict social functioning with younger people experiencing poorer social functioning than older people. This finding suggests the importance of controlling for age in examining relationships between psychological factors and levels of social functioning, and provides further support to the notion that mental health and social functioning problems begin during youth (Singh et al., 2010) and specifically in those with an ARMS (Cannon et al., 2008, Yung et al., 2004, Yung et al., 2005). However, whether the strength of the relationship varies with age cannot be determined from the study.

There are substantial limitations in this study. First, the data are cross-sectional in nature and, therefore, causality cannot be determined. Furthermore, the measures used were administered at post-treatment, and although we controlled for treatment we have no way of knowing how this might have affected the relationships observed. Further, the CT model used in EDIE-2 (French and Morrison, 2004) permits the targeting of metacognitive beliefs as well as cognitive schemas. The main aim of EDIE-2 was to reduce transition to psychosis, so it is expected that symptom severity would also be affected by the intervention. Therapists work with participants on identifying core beliefs (e.g. ‘I am worthless’), and metacognitive beliefs (e.g. ‘If I keep thinking in this way I will go mad.’). Beliefs were addressed using CT strategies such as, creating alternative reasons for events, examining evidence, advantages and disadvantages analysis or through use of behavioural experiments (French and Morrison, 2004). Working with beliefs may have contributed to reducing the presence or impact of such cognitive and metacognitive beliefs as well as symptoms, affecting the results of this study.

Finally, the proportion of unique variance explained by metacognitions was very small, which questions the clinical significance of the findings. It should however be acknowledged that the current test is quite stringent as metacognitions emerged after the control of several factors including the provision of treatment that might impact directly on social functioning

Despite these limitations, this study provides a preliminary indication that metacognitive beliefs about the uncontrollability and dangerousness of thoughts could be a predictor of social functioning in young people at risk for psychosis. Deconstructing this metacognitive belief by breaking it down into its constituent parts revealed that beliefs about the dangerousness of thoughts was of particular significance. Interventions for improving social functioning in those at risk for psychosis could consider targeting this metacognitive dimension. However, future longitudinal research should be conducted ensuring measures are administered in the pre-treatment phase. In doing this, we can perhaps come closer to understanding which psychological factors increase or reduce vulnerability to psychosis and poor social functioning as well as improving social recovery in an ARMS.

## Conflict of interest

None.
